# Development of a genetic tool for functional screening of anti-malarial bioactive extracts in metagenomic libraries

**DOI:** 10.1186/s12936-015-0748-6

**Published:** 2015-06-04

**Authors:** Alvaro Mongui, Francy J. Pérez-Llanos, Marcio M. Yamamoto, Marcela Lozano, Maria M. Zambrano, Patricia Del Portillo, Carmen Fernández-Becerra, Silvia Restrepo, Hernando A. Del Portillo, Howard Junca

**Affiliations:** RG Microbial Ecology: Metabolism, Genomics & Evolution - CorpoGen, Bogotá, Colombia; Department of Biological Sciences, Universidad de los Andes, Bogotá, Colombia; Departamento de Parasitologia, Universidade de São Paulo, São Paulo, Brazil; ICREA at ISGlobal, Barcelona Ctr Int Health Res (CRESIB), Hospital Clínic - Universitat de Barcelona, Barcelona, Spain; Institució Catalana de Recerca I Estudis Avançats (ICREA), Barcelona, Spain; Present Address: Applied Biology Program, Faculty of Basic & Applied Sciences, Universidad Militar Nueva Granada−UMNG, Campus Cajicá, Bogotá, DC Colombia

**Keywords:** Malaria, Metagenomics, Functional screening, Anti-malarial drugs, Synthetic gene design, Dermaseptin 4, Bioluminescent *Plasmodium falciparum*

## Abstract

**Background:**

The chemical treatment of *Plasmodium falciparum* for human infections is losing efficacy each year due to the rise of resistance. One possible strategy to find novel anti-malarial drugs is to access the largest reservoir of genomic biodiversity source on earth present in metagenomes of environmental microbial communities.

**Methods:**

A bioluminescent *P. falciparum* parasite was used to quickly detect shifts in viability of microcultures grown in 96-well plates. A synthetic gene encoding the Dermaseptin 4 peptide was designed and cloned under tight transcriptional control in a large metagenomic insert context (30 kb) to serve as proof-of-principle for the screening platform.

**Results:**

Decrease in parasite viability consistently correlated with bioluminescence emitted from parasite microcultures, after their exposure to bacterial extracts containing a plasmid or fosmid engineered to encode the Dermaseptin 4 anti-malarial peptide.

**Conclusions:**

Here, a new technical platform to access the anti-malarial potential in microbial environmental metagenomes has been developed.

## Background

Malaria is one of the most important and widely spread infectious diseases, especially in developing countries, constituting a major health threat to about 40 % of the world population [1]. The World Health Organization (WHO) estimated 207 million new cases of malaria worldwide in 2012, with over 600,000 deaths as a result of the severe complications associated with the disease. At present there is no fully effective vaccine against malaria, leaving chemotherapy as one of the most immediate options for fighting this disease [2]. Unfortunately, the number of available anti-malarial drugs with different chemotypes and novel mechanisms of action is low, and the number of compounds entering clinical trials is even lower [3], limiting the expected availability of new, approved compounds for human infections in the near future.

In addition, the rate at which drug resistance is emerging and expanding exceeds the current development of new drugs, in particular given the lengthy process from compound characterization to approval for use in humans [4]. This fact is of particular concern when taking into account the reports of artemisinin-resistant *Plasmodium falciparum* strains in Southeast Asia [5, 6]. Therefore, it is essential to keep searching for new anti-malarial chemotypes with novel mechanisms of action.

Efforts in recent years to find anti-malarial compounds have focused on the exploration of chemical libraries of public domain, using high-throughput screenings (HTS) on parasite culture [7–9]. Natural products, which have been the basis of the majority of current anti-malarial medicines [10], are also an important source of compounds of medical importance as they may include different structures for optimizing therapeutic measures against malaria. For this reason, research for the identification and characterization of new anti-malarial agents over the past years has gone beyond the study of plants to include marine organisms, fungi and bacteria [11].

Nowadays it is widely accepted that the microbial world, besides being the largest fraction of biodiversity on the planet, is one of the greatest sources for drug discovery, with frequent applications in human health [12]. It is very likely that the wide diversity of unexplored microbial functions may harbour several uncharacterized molecules, such as metabolites or peptides with potential therapeutic activity. Efforts to elucidate the genomes from non-cultured microorganisms (about 99 % of the total diversity), coupled with the need to discover compounds with biotechnological potential, has promoted the development of metagenomics [13]. This approach involves the direct extraction of total DNA from a given environment, which is subsequently cloned and transferred into bacterial hosts (so far, mainly in *Escherichia coli*) allowing the construction of metagenomic libraries. These libraries are often subjected to functional analysis for phenotypic identification of a particular activity.

The identification of anti-malarial compounds in metagenomic libraries is a promising alternative, but it represents additional technical challenges. Despite the success in identifying novel drugs from natural sources, pharmaceutical companies still prefer to carry out massive screens of pure synthetic compounds [14], an approach that is difficult in metagenomic libraries. For instance, the screening of gene libraries requires assessing parasitic cultures in the presence of complex bacterial clone extracts on which is hard to identify potential antiparasitic activity. To overcome this limitation, this research focused on the design and development of a non-radioactive methodology to screen metagenomic libraries for anti-malarial activity using bacterial extracts. To do this, different extracts expressing the anti-malarial peptide Dermaseptin S4 (DS4) [15] were tested in order to optimize the experimental conditions that promote antiparasitic activity detection against a novel *P. falciparum* bioluminescent strain. With this purpose, a platform for the parasite growth consisting of 96-well microplates was established, which allowed for both the incubation of multiple bacterial extracts and the efficient detection of the parasite’s viability, achieved in a multiple, reproducible and composite manner. The results gathered here pave the way for future mid- to large-scale analysis of metagenomic library clones, in a novel strategy for the expanded exploration of microbial diversity for anti-malarial traits by culture independent means.

## Methods

### Parasite cultures

The transgenic *P. falciparum* 3D7/pHDEF1-luc line [16] was grown continuously following the method of Trager and Jensen [17], under the selective pressure of drug WR99210 (Sigma-Aldrich, Saint Louis, USA). Parasites were cultured in complete medium consisting of RPMI 1640 with L-glutamine (Sigma), 25 mM HEPES (Sigma), 25 mM sodium bicarbonate (Sigma), 0.1 mM hypoxanthine (Sigma), 0.5 % Albumax II (Invitrogen, Carlsbad, USA), 50 μg/L gentamicin (Life Technologies, Carlsbad, USA) and human blood type O at 1.5–3 % haematocrit. The stock cultures were maintained in a 1–3 % parasitaemia range, under an atmosphere containing 5 % CO_2_, 5 % O_2_ and 90 % N_2_ at 37 °C.

### Isotopic and luminescence assays

The isotopic detection protocol used has been described by Desjardins *et al.* [18], except for the addition of the radioactive marker [3H]-labelled hypoxanthine. The assay was carried out in 96-well plates containing 130–200 μL of parasite culture at 1 % parasitaemia and 1 % haematocrit per well. For luciferase detection, parasite survival was evaluated after 48–72 h of drug or bacterial extract exposure and incubation at 37 °C. Briefly, the test plate was centrifuged and the supernatant discarded, while the remaining pellets were re-suspended in 10 μL of cell culture lysis buffer (10 mM Tris pH 7.5, 1 mM EDTA, 2 % Triton X-100 and 0.15 % saponin) and transferred to a 96-well, white, flat-bottom plate (Thermo Scientific, Waltham, USA). Then 10 μL of luciferase assay substrate (Promega, Fitchburg, USA) were added to each well, mixed for 60 s in a shaker and the luciferase activity (total light) measured in the TECAN GENios (Tecan, Männedorf, Switzerland) in terms of relative luminescence units (RLUs).

### Parasite growth inhibition tests

To perform inhibition tests, the parasite cultures were first synchronized with 5 % sorbitol (Sigma), as described elsewhere [19]. Parasite growth inhibition by drugs was tested using serial dilutions of chloroquine, mefloquine, artesunate, and amantadine. The 50 % inhibitory concentration (IC50) values were calculated from three different experiments in duplicates using the software LN-NonLin (V 1.05 Beta), based on a polynomial regression model. Parasite growth inhibition tests using bacterial extracts (see below) were done in triplicates using 8, 16 or 32 μL of each extract in 130 μL final volume per well, following the described procedures for parasite culture. Finally, the parasite growth ratio was determined according to the following formula: [(RLUs experimental sample - RLUs negative control)/(RLUs positive control - RLUs negative control)] × 100.

### Anti-malarial peptide selection

To select the peptide with anti-malarial activity to be used in this study, the literature was reviewed to find those that showed good antiparasitic activity in concentrations that were at least one order of magnitude lower than those required to have toxic effects on human cells. The peptide selection criteria included linear structure, a capacity to inhibit growth of *P. falciparum* by at least 50 % at concentrations below 20 μM, and activity detectable within a 72-h period. The peptide DS4 [15, 20] was chosen, being characterized by having a sequence of 38 amino acids and an IC50 range of 0.27–2.2 μM against *P. falciparum* strains sensitive and resistant to chloroquine.

### Dermaseptin 4 coding gene design

The DS4 coding sequence (CDS) was determined based on the more robust codons for *E. coli* K12 strain, using the peptide’s sequence (ALWMTLLKKVLKAAAKAALNAVLVGANA) [15] as a query in Optimizer [21]. The first codon (ATG) was included in the CDS. The arabinose promoter (P_BAD_) and the ribosomal RNA B transcriptional termination region, both from pBAD18 plasmid, were included *in silico* upstream and downstream on DS4 CDS, respectively [22]. Secondary structure prediction of the theoretical mRNA sequence was performed by Mfold [23], choosing only the −4 to +37 pair bases around the ATG. The complete designed gene was chemically synthesized (Genscript, Piscataway, USA) and subsequently cloned in the unique *Eco*RV restriction site of pUC57 plasmid (pUC57_DS4).

### Synthetic gene cloning and RT-PCR

With pUC57_DS4 as template full *ds4* gene amplification was performed using the following primers: 5′-ggcgcgccACTTTTCATACTCCCGC-3′ and 5′-ggcgcgccTGTATCAATAAAACGAAAGG-3′ (*Asc*I restriction sites shown in lower case). The amplified product was cloned into the unique *Asc*I restriction site of pCC2FOS_F076 (F076), a fosmid containing a metagenomic insert obtained from soil DNA libraries of potato agricultural plots in Colombian Andes (Proyecto CIMA, Ministerio de Agricultura, República de Colombia). The fosmid DNA sequence was obtained by 454 technology (Selah Genomics, Greenville, USA) (fosmid sequence data assembled and kindly provided by JC García-Betancur, Molecular Biotechnology - CorpoGen & IMIB - U. Würzburg). The final DNA construct (clone F076_DS4) was confirmed by sequencing.

To assess *ds4* transcription from the synthetic gene, RNA was extracted from *E. coli* Epi300 bacterial clones (Illumina Inc, San Diego, USA) harbouring one of the following vectors: pUC57 (empty plasmid vector), pUC57_DS4, pCC2FOS (empty fosmid vector), pCC2FOS_F076 or pCC2FOS_F076_DS4. Briefly, each bacterial clone was grown to 0.4–0.8 OD_600nm_ in LB medium with chloramphenicol (20 μg/mL) or ampicillin (100 μg/mL), depending on the vector backbone of each episomal DNA. Peptide expression was repressed or induced, respectively, with 0.1 % D-glucose or 0.2 % L-arabinose for four additional hours at 37 °C. RNA extraction was performed on every cell pellet using the Direct-zol RNA MiniPrep kit (Zymo Research, Irvine, USA). RNA samples were treated with RQ1 RNase-Free DNase (Promega), quantities normalized, and then subjected to reverse transcription using 15 ng/μL of random hexamers and SuperScript III Reverse Transcriptase (Invitrogen), following the manufacturer’s recommendations. Subsequently, the coding length for the DS4 peptide was amplified by PCR using the primers DS4-F (5′-ATGGCCCTGTGGATGACCCT-3′) and DS4-R (5′-TCAGGCGTTGGCACCG-3′).

### Peptide expression and whole protein extraction

To obtain whole bacterial extracts post induction with L-arabinose, transformed *E. coli* Epi300 cells were grown as explained above. Bacterial cultures were normalized by optical density (OD) and their respective pellets were washed three times with PBS, re-suspended in 1/20 of the original volume in PBS and lysed by sonication for 2 min in a Qsonica Q500 sonicator (Cole-Parmer, Vernon Hills, USA), using 1 s pulse, 1 s rest and 12 % amplitude. Resulting lysed cultures were filtered (0.22 μm), quantified using the Bradford assay [24], and normalized based on the extract that showed the lowest soluble protein concentration. All the bacterial extracts were assessed on *P. falciparum* 3D7/pHDEF1-luc culture as described.

## Results

The development of a stable *P. falciparum* 3D7 transgenic line, capable of expressing the firefly luciferase gene under the control of the intergenic region of *Plasmodium berghei* elongation factor 1 (EF-1α) gene has been previously reported [16]. Moreover, a remarkable sensitivity of the luciferase assay using this transgenic parasite and a well-correlated linear relationship between luminescence and parasite density (r = 0.98) has been observed. *Plasmodium falciparum* 3D7/pHDEF1-luc was therefore used to develop a method for bioluminescence detection in 96-well microplates by assessing if parasite bioluminescence, as a determinant of parasite growth, was comparable to standard radioactive detection methods [18, 25]. Four anti-malarial drugs with different mechanisms of action were tested *in vitro* against *P. falciparum* 3D7/pHDEF1-luc cultures. Results demonstrated that IC50 values using the luciferase assay were not significantly different from the ones using the hypoxantine method (Table 1). The high variability observed in the triplicates of mefloquine is not uncommon in these assays [26]. Therefore, this transgenic parasite can be used to develop mid- to high-throughput tests for identifying potential anti-malarial compounds, as already shown for other bioluminescent transgenic lines similarly constructed [9, 27].

**Table 1:** IC50 comparison (mean ± SD) for the anti-malarial compounds tested by two detection methods

Detection method	Anti-malarial drug			
	Chloroquine (nM)	Mefloquine (nM)	Artesunate (nM)	Amantadine (μM)
Isotopic	10.18 ± 2.37	17.28 ± 8.38	1.05 ± 0.57	112.31 ± 24.03
Luciferase	10.36 ± 0.75	15.83 ± 7.40	0.81 ± 0.47	132.24 ± 43.08

**Fig. 1:**
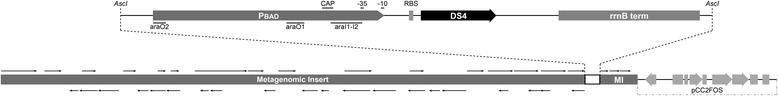
DS4 coding gene and F076_DS4 construct development. Scaled diagram of the gene designed to control DS4 anti-malarial peptide expression from the arabinose inducible promoter (P_BAD_). The final construction in fosmid F076_DS4 harboring the *ds4* gene in the metagenomic insert is also shown. araO2, araO1 and araI1-I2: promoter regulator regions; CAP: catabolite activator protein binding site; RBS: ribosome binding site; rrnB term: RNA B transcriptional termination region; MI: metagenomic insert; pCC2FOS: fosmid vector. Horizontal black arrows represent all open reading frames (ORFs) predicted in the MI with a minimum length of 450 pb, that started only with ATG

The DS4 peptide was chosen for the design of a gene capable of promoting anti-malarial activity when expressed in *E. coli*. This linear peptide, isolated from the skin glands of *Phyllomedusa sauvagii* frogs, has shown anti-malarial effect (evidenced by radiolabeled hypoxanthine parasite incorporation) on both chloroquine-sensitive and resistant *P. falciparum* strains, with IC50 values ranging from 0.27 to 2.2 μM [15]. The coding sequence for DS4 peptide was designed based on the most robust *E. coli* codons, different from the common approach of using the most frequent codons or those which optimize the codon adaptation index (CAI) [28]. This has been shown to be a determinant for efficient expression of some recombinant proteins, primarily determined by the availability of the amino-acylated (charged) tRNAs and not by the total levels of tRNAs [29]. Transcription of this coding sequence is controlled by an upstream ribosome-binding site (RBS) and the arabinose promoter (P_BAD_), and a downstream transcriptional terminator region (Fig. 1). The *in silico* identification of secondary structures in the region −4 to +37 (from the first ATG codon) showed a single secondary structure with a free energy value of −7.5 kcal/mol, much higher than the values reported as detrimental for an efficient initiation of recombinant protein synthesis in *E. coli* [30].

**Fig. 2:**
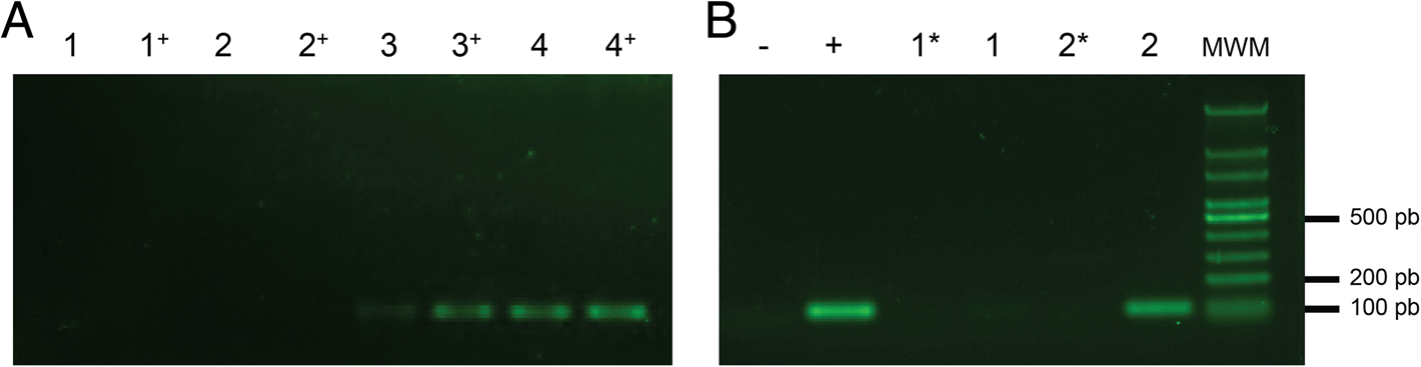
DS4 coding gene transcription from different episomal DNAs. **a** DS4 CDS amplification from cDNAs of bacterial cultures grown in the presence or absence of L-arabinose: 1 - *E. coli* Epi300 pCC2FOS_F076; 2 - *E. coli* Epi300 pUC57; 3 - *E. coli* Epi300 F076_DS4; 4 - *E. coli* Epi300 pUC57_DS4. +, indicate the cultures that were induced with L-arabinose, while the rest of the samples did not include the inducer. **b** DS4 CDS amplification from cDNAs from *E. coli* Epi300 F076_DS4, after being incubated with the repressor (D-glucose) or the inductor (L-arabinose) of gene transcription. -, PCR mix without DNA; +, positive control for the PCR (with pUC57_DS4 as a template); 1, cDNA from bacterial culture incubated with D-glucose; 2, cDNA from bacterial culture incubated with L-arabinose; *, RT-PCR reactions without reverse transcriptase; MWM, molecular weight marker

Once the *ds4* synthetic gene was obtained, it was amplified and inserted into a unique restriction site of the metagenomic insert of pCC2FOS_F076. After transforming both episomal DNAs harbouring the *ds4* gene (pUC57_DS4 and F076_DS4) in *E. coli* Epi300, gene transcription was validated by independent induction of bacterial gene expression with L-arabinose, compared with the same DNAs lacking the *ds4* gene (Fig. 2a). The P_BAD_ was able to both promote *ds4* gene transcription from the F076_DS4 construct (in the context of metagenomic DNA), and to tightly repress the *ds4* transcription by incubating the bacterial culture with D-glucose (Fig. 2b). Since there are no previous reports of recombinant DS4 peptide expression it is important to mention that the regulatory system used in this research, based on the interaction of P_BAD_ with the AraC protein, has proven to be quite useful for the expression of potentially toxic proteins [22]. Although the AraC protein is essential for transcription from P_BAD_ [31], its encoding gene was not included in the synthetic *ds4* gene. This is due to the fact that the *E. coli* Epi300 strain used has a genomic copy of the *araC* gene that supplies that regulatory function [32]. Therefore, DS4 peptide expression from the designed synthetic gene proposed in this research is restricted to bacterial hosts that express the AraC protein.

**Fig. 3:**
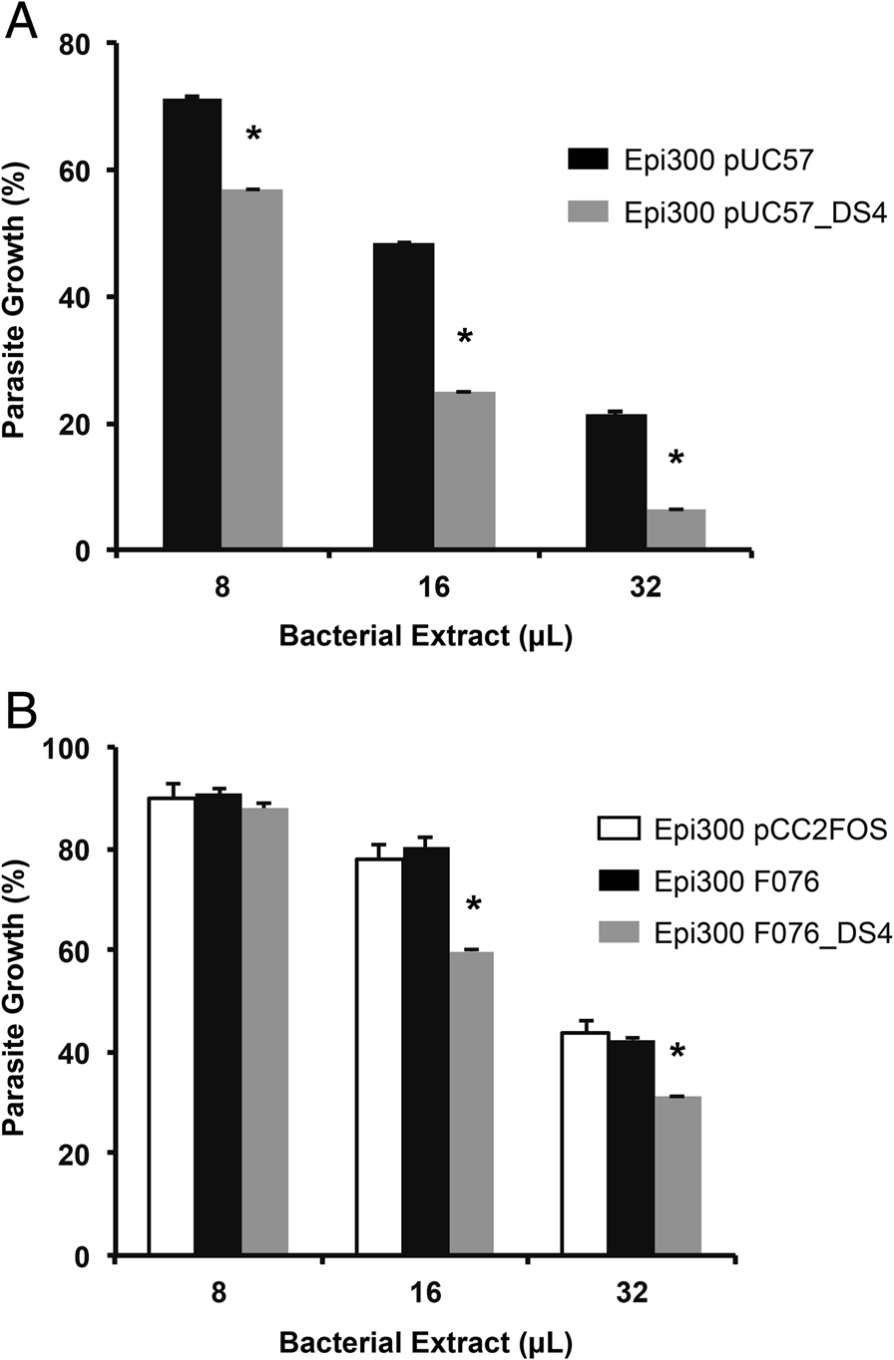
Anti-malarial activity with extracts from bacterial pellets. Parasite growth inhibition upon incubation of the transgenic *P. falciparum* 3D7/pHDEF1-luc with different volumes of L-arabinose-induced *E. coli* Epi300 extracts. **a** Parasite growth inhibition with extracts derived from *E. coli* Epi300 transformed with pUC57 or pUC57_DS4. **b** Parasite growth inhibition with extracts derived from *E. coli* Epi300 transformed with pCC2FOS, pCC2FOS_F076 (F076) or F076_DS4. Error bars represent SD; *, p <0.05 by paired Student’s t-test

Parasitic growth inhibition assays were performed using the cell pellet extracts (concentrated samples) from *E. coli* Epi300 pUC57_DS4 and the metagenomic clone *E. coli* Epi300 F076_DS4, compared with the controls not expressing the DS4 peptide. The DS4 peptide is lipophilic and has high electrostatic affinity for cell membranes [33], a partition that would result in a relative decrease of its concentration in the soluble fraction, after using the non-denaturing extraction protocol proposed. After standardizations, significant anti-malarial activity was observed using bacteria expressing the DS4 peptide from plasmid and fosmid DNAs (Fig. 3), demonstrating the feasibility of this approach. It is important to note that the bacterial pellet extracts from *E. coli* harbouring either one of the episomal DNAs lacking the antimalarial DS4 peptide coding gene (pUC57, pCC2FOS or pCC2FOS_F076) had *per se* an inhibitory growth effect on the parasite proportional to the amount of bacterial extract used. Thus, it is necessary to normalize bacterial extract concentration when testing multiple metagenomic clones, in order to observe antiparasitic activity over the inhibitory effect of the extract itself, avoiding false positives. These results showed that bacterial extracts expressing the recombinant anti-malarial DS4 peptide exerted overall significantly higher parasite mortalities when compared to the effect of the extracts alone. This system can be used to screen bacterial extracts with metagenomic-encoded information for anti-malarial activities using the tested and optimized bioluminescent assay for *P. falciparum* viability in a 96-well format.

## Conclusions

Here, the development of a novel screening platform for exploring novel anti-malarial activities encoded in the information held in metagenomic libraries is reported. The platform involved the establishment of malarial parasite cultures in 96-well plates using a novel *P. falciparum* bioluminescent strain and the construction of a bacterial clone capable of expressing a peptide with anti-malarial activity in the context of whole bacterial extracts. This bacterial clone serves as a positive control for anti-malarial activity in current and future analyses of clones from genomic/metagenomic libraries of diverse and scarcely explored sources. The combined use of these components defined a tool aiming to facilitate the retrieval of novel anti-malarial compounds from metagenomic libraries containing information originated from diverse microbial communities as potential novel sources for chemotherapy developments. However, future assays should be focused on increasing the metagenomic clone screening rates. One approach could include the assessment of antimalarial activities by bacterial extract pools or in 384-well plate format. Alternatively, metagenomic libraries with improved functional expression profiles (e.g. from multiple bacterial hosts and libraries constructed in vectors including strong-flanking promoters) or containing high proportion of microorganisms more related to the screening host may be appropriate starting points for antimalarial-research studies from metagenomic libraries.

## Abbreviations

HST: High-throughput screenings
DS4: Dermaseptin 4
CDS: Coding sequence
RLUs: Relative luminescence units
CAI: Codon adaptation index
RBS: Ribosome-binding site
